# Predicting flexural strength of hybrid FRP-steel reinforced beams using symbolic regression and ML techniques

**DOI:** 10.1038/s41598-025-05775-7

**Published:** 2025-06-25

**Authors:** Khaled Megahed

**Affiliations:** Department of Structural Engineering, Mansoura University, PO BOX 35516, Mansoura, Egypt

**Keywords:** Fiber-reinforced polymer, Flexural strength, Hybrid FRP-steel reinforced beams, Machine learning, Symbolic regression, CatBoost model, Civil engineering, Scientific data, Composites, Statistics

## Abstract

Hybrid fiber-reinforced polymer (FRP) and steel reinforced concrete (hybrid FRP-steel RC) beams have gained recognition for their exceptional flexural performance, surpassing that of beams reinforced exclusively with FRP bars (FRP-RC). However, current design guidelines, such as ACI 440.11–22, fail to accurately predict the flexural strength of these hybrid systems. This study aims to enhance the predictive accuracy and interpretability of flexural strength models by applying advanced computational approaches—specifically, machine learning (ML) techniques and symbolic regression. A robust dataset of 134 experimental data points was utilized to develop predictive models. The prediction results showed that both ML and symbolic regression models significantly outperformed the ACI 440.11–22 equations, achieving lower errors (MAE, MAPE, RMSE) and higher accuracy (R^2^). The results demonstrate that the ML models—Gaussian process regression (GPR), NGBoost, and CatBoost—achieved high predictive accuracy, with mean R^2^ values approaching 1.0 and MAPE% as low as 5.19 (training) and 11.51 (testing) for GPR. Furthermore, symbolic regression yielded a transparent mathematical expression with a mean prediction ratio (µ) of 1.003, a CoV of 0.139, and a MAPE% of 11.08. These findings highlight the practical and technical advantages of symbolic regression in developing reliable, interpretable, and efficient design equations for hybrid FRP-steel RC beams.

**Supplementary Information:**

The online version contains supplementary material available at 10.1038/s41598-025-05775-7.

## Introduction

Fiber-reinforced polymers (FRPs) have gained attention as a corrosion-resistant substitute to traditional steel reinforcement in concrete buildings, offering a solution to the durability limitations of conventional steel-reinforced concrete (steel-RC) members^1^. However, the widespread adoption of certain FRP bars, such as glass FRP (GFRP) and basalt FRP (BFRP), is hindered by their low elastic modulus and linear stress-strain behavior. These properties often result in brittle failure modes, excessive crack widths, and increased deflections in FRP-reinforced concrete (FRP-RC) members compared to their steel-RC counterparts with equivalent reinforcement ratios^2^. To address these challenges, a novel approach has emerged: the use of hybrid reinforcement systems combining steel and FRP bars. In this configuration, steel rebars are strategically placed in the inner layer, while FRP bars are positioned near the outer surface of the tensile zone. This hybrid configuration not only improves structural performance but also enhances ductility and reduces deflections, offering a balanced solution for modern structural design^3–5^.

Hybrid reinforcement systems have attracted considerable research attention in recent years. The studies by Ruan et al.^3^, Huessin et al.^4^, and Wang et al.^5^ have explored the flexural behavior of hybrid RC beams. Their findings consistently demonstrate the effectiveness of hybrid reinforcement in improving the ductility and stiffness of FRP-RC beams. Hybrid FRP-steel RC beams typically exhibit greater flexural capacity than steel-RC beams with equivalent reinforcement areas, while also providing enhanced stiffness and ductility compared to pure FRP-RC beams^6,7^. This is attributed to the high tensile strength of FRP bars, which boosts the ultimate load-bearing capacity, and the plasticity and high modulus of steel reinforcement, which contribute to ductility and stiffness.

The flexural strength of concrete beams with hybrid reinforcement is typically determined using theoretical frameworks derived from principles applicable to steel-RC or FRP-RC structures. Qu et al.^8^ have developed calculation formulas for the flexural capacity of hybrid FRP-steel RC beams. Following this, different formulations tailored for hybrid FRP-steel RC beams have been proposed^7,9–11^. According to the ACI 440.11–22^12^ guidelines, FRP-RC beams exhibit a transition region where the failure mode becomes uncertain, potentially manifesting as either compression or tension failure. This ambiguity arises when the FRP reinforcement ratio falls between 0.75 and 1.4 times the balanced FRP reinforcement ratio due to the variations in the actual strengths of concrete and FRP materials. The ACI 440.11–22^12^ guidelines continue to use the balanced FRP reinforcement ratio as the threshold for distinguishing between tension and compression failure modes when predicting the nominal flexural strength of FRP-RC beams. However, a significant limitation of these guidelines is their reduced accuracy in calculating the flexural strength of hybrid FRP-steel RC beams within this transition region^11^.

Experimental studies are frequently conducted to understand the behavior of hybrid FRP-steel RC beams^13–15^. However, these investigations are often constrained by limited parameter ranges and can be both expensive and time-consuming. Other researchers have employed finite element and numerical analytical methods to simulate the flexural behavior of hybrid FRP-steel RC beams, providing deeper insights into the underlying physics of these structural phenomena^16,17^. On the other hand, machine learning (ML) techniques have emerged as valuable tools to complement experimental work, demonstrating considerable success in predicting the behavior of structural elements. Researchers have employed ML algorithms such as Gaussian Process Regression (GPR)^18^, artificial neural network (ANN)^11^, gene expression programming (GEP)^19^, and symbolic regression (SR)^20^ to develop statistical models and empirical formulas for predicting the performance of structural members. Numerous studies have applied ML techniques to predict key structural properties of composite members, such as the shear strength of FRP-RC beams without reinforcement^21^, the axial strength of composite columns^22^, the shear and flexural strengths of ultra-high performance concrete^23,24^, and bending capacity of ECC-concrete composite beams^25^. For example, Katlav and Ergen^26^ used a CatBoost model optimized with various metaheuristic algorithms to accurately predict the compressive strength of ultra-high-performance concrete, demonstrating the strong potential of ML in forecasting material behavior. These studies collectively demonstrate the accuracy and reliability of ML techniques in predicting the mechanical and structural properties of concrete.

Furthermore, symbolic regression is a powerful machine learning technique that focuses on discovering mathematical expressions or equations that best describe the relationships within a dataset. Unlike traditional regression methods that assume a predefined model structure, symbolic regression explores a wide range of possible equations, making it highly flexible and capable of uncovering complex, interpretable relationships. Symbolic regression is closely linked to genetic programming (GP) and gene expression programming (GEP), as both are evolutionary algorithms used to evolve mathematical expressions. Symbolic regression leverages the principles of GP and GEP to evolve optimal mathematical models that fit the data, combining the strengths of evolutionary computation with the interpretability of symbolic models. For example, Megahed et al.^20^ implemented SR model with various ML models for predicting the shear strength of deep beams. Similarly, Zhang et al.^11^ developed a GEP model for predicting the flexural strength of concrete beams reinforced with hybrid FRP and steel bars. Furthermore, Tarawneh et al.^27^ generated a mathematical GEP model for designing slender FRP-RC columns.

This study focuses on developing two advanced computational approaches—machine learning (ML) and symbolic regression—to predict the flexural strength of hybrid FRP-steel reinforced concrete (RC) beams. Geometric characteristics and material parameters, which are critical parameters of flexural strength, were used to construct and train the ML and symbolic regression models. The performance of the proposed ML and symbolic regression models was compared against the predictions of the ACI 440.11–22^12^ standards.

### Dataset description

**Fig. 1:**
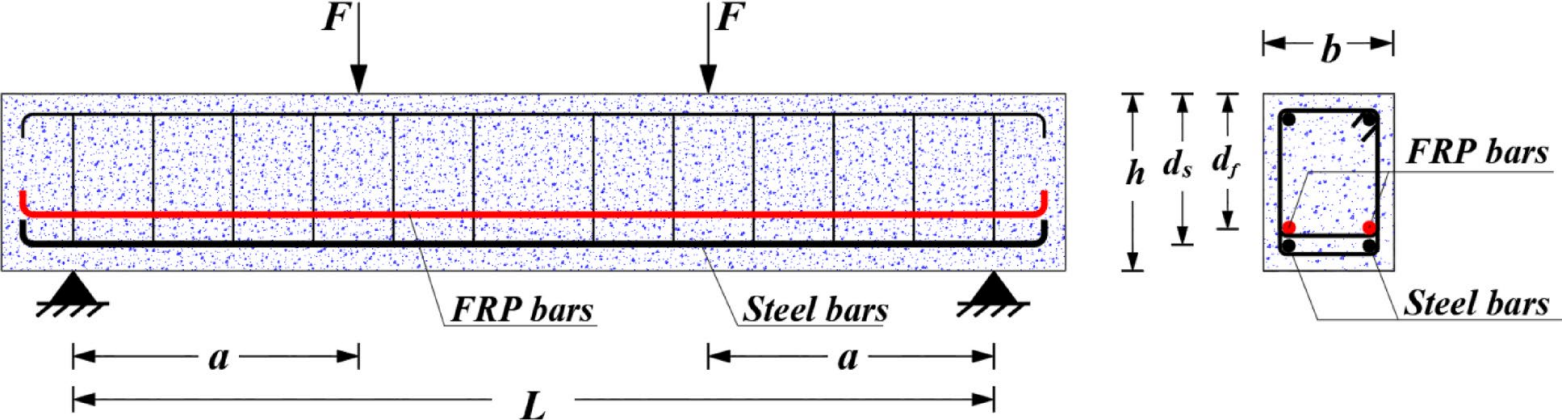
General geometry of hybrid FRP-steel RC beams.

To develop machine learning (ML) and symbolic regression models for predicting the flexural behavior of hybrid FRP-steel reinforced concrete (RC) beams, a comprehensive experimental dataset comprising 134 beam specimens collected by Zhang et al.^11^ is implemented. As illustrated in Fig. 1, the configuration of these hybrid beams includes both steel and FRP reinforcement. Table 1 summarizes the key details and the statistical distributions of the specimens, including: beam width ($\:b$), beam depth ($\:d$), steel reinforcement ratio ($\:{\rho\:}_{s}$%​), FRP reinforcement ratio ($\:{\rho\:}_{f}$%), steel bar yield strength ($\:{f}_{y}$), concrete compressive strength ($\:{f}_{c}$), elasticity modulus of FRP bars ($\:{E}_{f}$​), ultimate tensile strength of FRP bars ($\:{f}_{fu}$​), and the beam flexural strength ($\:{M}_{u}$). The output variable chosen in this study is the dimensionless strength index, denoted as *m*_*u*_. This index is computed by normalizing the flexural moment strength *M*_*u*_ as follows:

**Table 1:** Summary of hybrid FRP-steel RC beams database. # is the number of experimental tests for each reference.

Ref	#	$\:b$ (mm)	$\:d$ (mm)	$\:{\rho\:}_{s}\%$	$\:{\rho\:}_{f}\%$	$\:{f}_{c}^{{\prime\:}}$ (MPa)	$\:{f}_{y}$ (MPa)	$\:{f}_{fu}$ (MPa)	$\:{E}_{f}$ (GPa)	$\:{m}_{u}$
^42^	3	175–200	350–400	0.23–0.53	0.04–0.09	30.0	324	1775.0	52.0	0.75–0.94
^43^	4	150	200	0.45-1.0	0.34–0.9	45.7	465	1366–1674	49.0–50.0	0.42–0.65
^44^	4	150	200	0.81	0.59–0.89	28.5–48.8	460	760	40.8	0.49–0.62
^8^	6	180	250	0.29–3.53	0.36-1.0	26.5–32.5	336–363	755–782	37.7–45.0	0.36–0.62
^6^	3	280	380	0.66–1.03	0.3–1.03	39.8–44.6	336–597	582–588	38.0-39.5	0.69–0.78
^7^	3	200	300	0.6–0.91	0.39–0.58	28.1	360	880.0	55.0	0.66–0.7
^45^	3	150	250	2.94–3.33	0.71–0.86	64.0	375	1301	75.98	0.38–0.47
^9^	3	200–220	300–350	0.1–0.44	0.09–0.38	35.0-44.2	400–511	1141–1373	45.7–55.0	0.68–1.07
^46^	6	200	350	0.1–0.4	0.09–0.16	44.2	423–580	1246–1373	55.0	0.97–1.09
^16^	7	200	450	0.06–0.2	0.06–0.12	56.0	456–599	1240–1520	63.5–83.8	0.73–0.94
^47^	2	230	250	1.81	0.27–0.8	75.9	470	941–2130	48.1–146	0.49–0.53
^48^	4	100	200	0.42–0.74	0.88–1.27	30.0	470–530	755–780	39.0–41.0	0.37–0.46
^13^	6	230	300	0.15–0.72	0.44–0.72	40.0	520–650	1000	50.0	0.39–0.59
^49^	1	180	250	1.03	0.26	30.2	450	1210	46.0	0.53–0.53
^50^	6	150	200	0.30–0.86	0.19–0.58	30.2	340–507	1250	50.0	0.44–0.79
^51^	3	200	300	0.40–0.95	0.78–1.17	31.0	500	1184	62.6	0.43–0.62
^52^	3	220	300	0.40–0.47	0.40–0.40	34.4	400	1145	49.0	0.62–0.68
^53^	12	150	250	0.54–1.06	0.36–1.19	29.9–35.4	412	970	44.3	0.44–0.75
^54^	4	150	250	0.58–0.58	0.44–0.79	40.9–61.7	446	1118–1655	48.0-103.0	0.48–0.85
^3^	6	180	300	0.44–0.56	0.49–0.87	30.3	517–540	868–958	40.1–45.7	0.34–0.68
^14^	2	150	250	0.09–3.49	0.04–2.36	30.5–34.2	309–410	970	44.3	0.24–1.1
^55^	8	125	250	0.53–1.05	0.53–1.19	20.0	375	788–2070	43.9–124.0	0.22–0.6
^15^	17	200	300	0.09–1.09	0.21–1.05	30.5–31.3	470	449–1034	35.0–46.0	0.48–1.11
^5^	5	150	250	0.27–0.53	0.3–1.03	37.6–37.6	478–495	1077–1220	51.0	0.44–0.68
^56^	5	150	250	1.13–2.93	1.83–2.9	29.9–35.4	444–451	871–1142	47.3–53.1	0.14–0.27
^57^	5	180	250	0.58–1.61	0.58–1.61	32.6	540	1200	55.0	0.42–0.57
^4^	3	150	200	0.25–0.28	1.0-1.09	30.0	385	1100	48.0	0.3–0.31

1 $$\:{m}_{u}=\frac{{M}_{u}}{{A}_{f}{f}_{fu}{d}_{f}+{A}_{s}{f}_{y}{d}_{s}}$$

where $\:{A}_{s}$ is the steel bar area, $\:{A}_{f}$ is the FRP bar area, $\:{d}_{s}$ is the effective depth of steel bar, and $\:{d}_{f}$ is the effective depth of FRP bar. The strength index, *m*_*u*_, can capture the contribution of concrete strength and reinforcement ratios on the beam strength. It has been demonstrated that using the above normalized strength index instead of member strength as the primary output significantly improves machine learning prediction performance^20,28^.

**Fig. 2:**
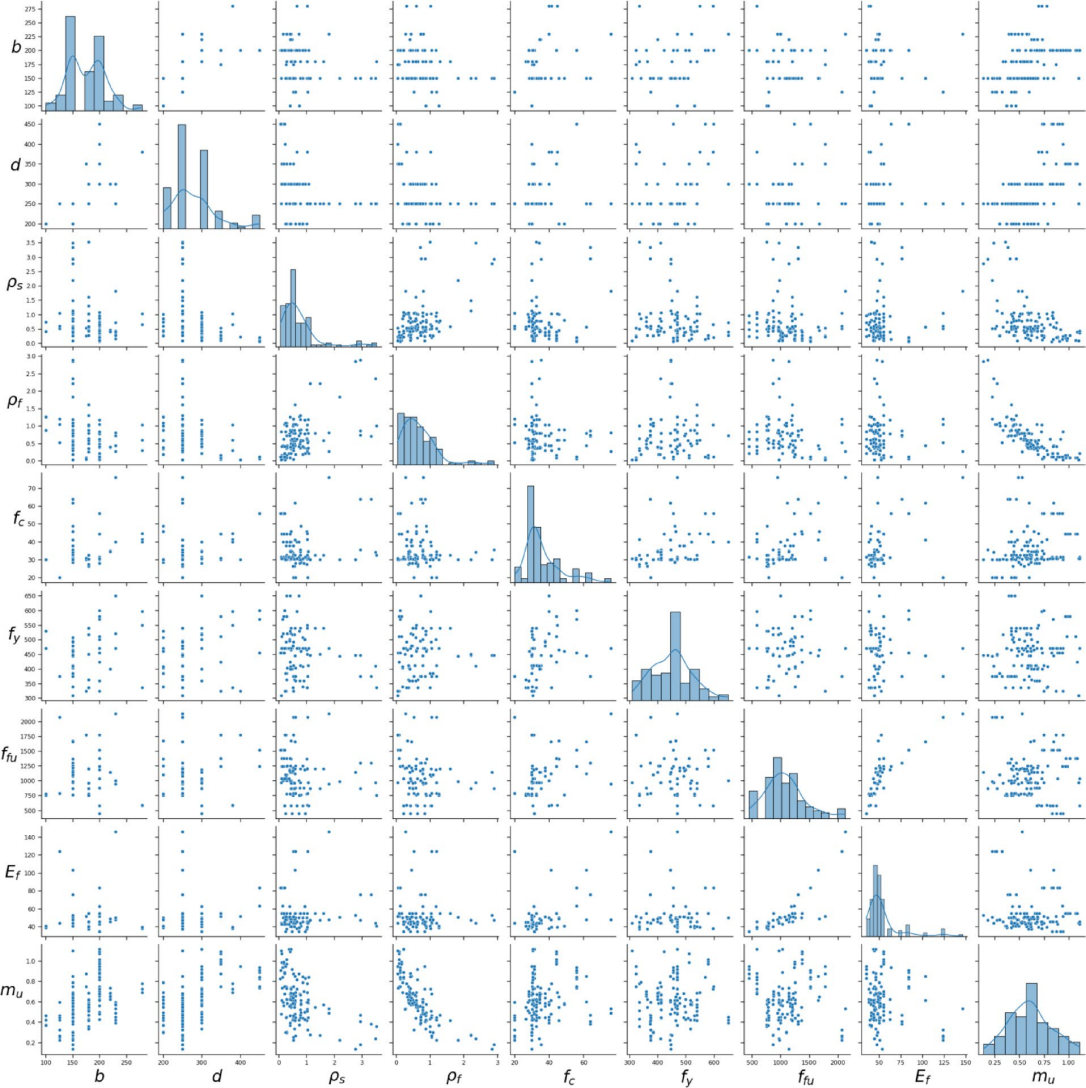
Distribution of the database and the relationships between different parameters.

Table 2; Fig. 2 provide statistical summaries of the output variable and the eight input features derived from the beam database. Beams with large values of the steel reinforcement ratio ($\:{\rho\:}_{s}$%​), FRP reinforcement ratio ($\:{\rho\:}_{f}$%) display small strength index due to the tendency of the beam to compression failure as indicated in (Fig. 2).

**Table 2:** Statistic features of the experimental dataset.

Variable	Symbol	Type	Statistics					
			Min	Max	Mean	Std	Skewness	Kurtosis
Beam width	$\:b$ (mm)	Input	100	280	175.9	35.1	0.345	0.29
Beam depth	$\:d$ (mm)	Input	200	450	278.3	60.6	1.114	1.32
Steel reinforcement ratio (%)	$\:{\rho\:}_{s}\%$	Input	0.06	3.53	0.73	0.68	2.47	6.40
FRP reinforcement ratio (%)	$\:{\rho\:}_{f}\%$	Input	0.038	2.90	0.67	0.51	1.87	4.97
Concrete strength	$\:{f}_{c}^{{\prime\:}}$ (MPa)	Input	20	75.9	36.0	10.8	1.50	2.39
Steel strength	$\:{f}_{y}$ (MPa)	Input	309	650	452	72.1	0.37	0.0015
FRP strength	$\:{f}_{fu}$ (MPa)	Input	449	2130	1083	353.4	0.79	0.84
FRP elasticity modulus	$\:{E}_{f}$ (GPa)	Input	35	146.2	53.3	19.24	2.72	7.72
Moment strength	$\:{M}_{u}$(kN.m)	--	5.85	261	56.38	33.52	2.81	13.75
Flexural strength index	$\:{m}_{u}$	Output	0.135	1.11	0.61	0.22	0.27	-0.34

### ML algorithms

This study employs six different machine learning (ML) models to predict the flexural strength of hybrid FRP-steel RC beams: categorical boosting (CatBoost)^29^, extreme gradient boosting (XGBoost)^30^, light gradient-boosting machine (LightGBM)^31^, natural gradient boosting (NGBoost)^32^, random forests (RF)^33^, gaussian process regression (GPR)^18^, and support vector regression (SVR)^34^. The performance of these models is evaluated and compared. Typically, ensemble learning methods offer greater accuracy and stability compared to individual models^29^.

CatBoost, LightGBM, NGBoost, and XGBoost are ensemble techniques based on boosting, where multiple weak learners are combined iteratively to create a stronger predictor^35^. CatBoost is particularly effective with categorical data, as it eliminates the need for preprocessing non-numerical features^29^. It uses unbiased boosting to reduce gradient bias and improve generalization, especially when working with categorical variables. LightGBM^31^ adopts a histogram-based approach for data splitting, making it faster and better suited for large datasets. NGBoost^32^, a gradient boosting-based algorithm for probabilistic prediction provides full probability distributions, enabling predictive uncertainty estimation. XGBoost^30^, on the other hand, uses a level-wise depth-first strategy, which may be slower than LightGBM but can yield more robust results for specific tasks. Random Forests, introduced by Breiman^33^, is an ensemble method based on bagging. It trains multiple decision trees on different subsets of data and aggregates their outputs through averaging (for regression) or voting (for classification). Important parameters affecting RF performance include the number of trees, maximum features, and tree depth.

In this study, the min-max scaling technique in the sklearn package is applied for data normalization to mitigate challenges arising from multidimensionality. Although tree-based models like CatBoost and LightGBM are scale-invariant, all input features were normalized to maintain consistency across models. After normalization, the dataset was randomly divided into training and testing subsets. To identify the most effective configuration for ML models, several training–testing split ratios (50–50%, 60–40%, 70–30%, 80–20%, and 90–10%) were examined. Among these, the 80–20% split produced the best performance, and was therefore selected to ensure optimal accuracy in the predictive analyses.

The performance of machine learning (ML) models heavily depends on the selection of hyperparameters, which must be defined prior to training. To achieve optimal predictive accuracy, it is essential to conduct hyperparameter tuning by exploring various parameter combinations. Traditional methods such as grid search (GS) and random search (RS) perform this exploration either exhaustively or randomly, often resulting in high computational cost, especially when dealing with models that have many hyperparameters and large search spaces. To overcome these limitations, this study employs Bayesian Optimization (BO) as an efficient strategy for hyperparameter tuning. BO uses a surrogate model—a probabilistic model that approximates the objective function—to predict the performance of hyperparameter configurations and guide the search process more intelligently. In this study, the Tree-structured Parzen Estimator (TPE)^36^, a non-parametric surrogate model known for its robustness and computational efficiency, was selected for BO. This choice enables the optimization algorithm to focus evaluations on promising regions of the search space while reducing redundant trials. Compared to GS and RS, BO with TPE significantly improves efficiency by requiring fewer iterations to locate optimal configurations^37^. To ensure robust performance and minimize overfitting, a 5-fold cross-validation strategy was applied during the hyperparameter tuning process. For each trial, the average Mean Absolute Percentage Error (MAPE) across the five folds was used as the optimization objective. The hyperparameter tuning process was implemented using the Optuna optimization framework, while the ML models themselves were developed using the scikit-learn, XGBoost, LightGBM, NGBoost, and CatBoost libraries. The value ranges and optimal hyperparameters for each model are presented in (Table 3).

**Table 3:** The optimal hyperparameters for ML models.

ML model	Optimal hyperparameters
CatBoost	iterations = 1936, learning_rate = 0.098, depth = 11, subsample = 0.156, colsample_bylevel = 0.071, min_data_in_leaf = 97
GPR	Kernel: Constant*RBF + Constant*Matern + Constant*WhiteKernel + Constant* RationalQuadratic, gpr.alpha = 0.001
LightGBM	n_estimators = 1562, learning_rate = 0.14, max_depth = 30, num_leaves = 10, boosting_type=’dart’
XGBoost	random_state = 1000, n_estimators = 791, max_depth = 39, learning_rate = 0.2, booster=’gbtree’, gamma = 0.01
RandomForest	random_state = 1000,n_estimators = 136, max_depth = 38, min_samples_leaf = 2, max_features=’sqrt’, bootstrap = False
NGBoost	Dist = Normal, Score = MLE, n_estimators = 505, learning_rate = 0.039, minibatch_frac = 0.34, natural_gradient = True

### Symbolic regression

Symbolic regression (SR)^38,39^ is a genetic programming technique designed to discover simple, interpretable equations that best represent a given problem by exploring a predefined space of mathematical expressions^19^. It is considered a multi-objective optimization problem, balancing the trade-off between predictive accuracy and model complexity. Using natural selection and evolution principles, SR iteratively refines candidate mathematical models, searching for the most satisfactory solutions. In this study, the Python library PySR^40^ is employed to identify concise and interpretable expressions for predicting the bending strength of hybrid FRP-steel RC beams.

**Fig. 3:**
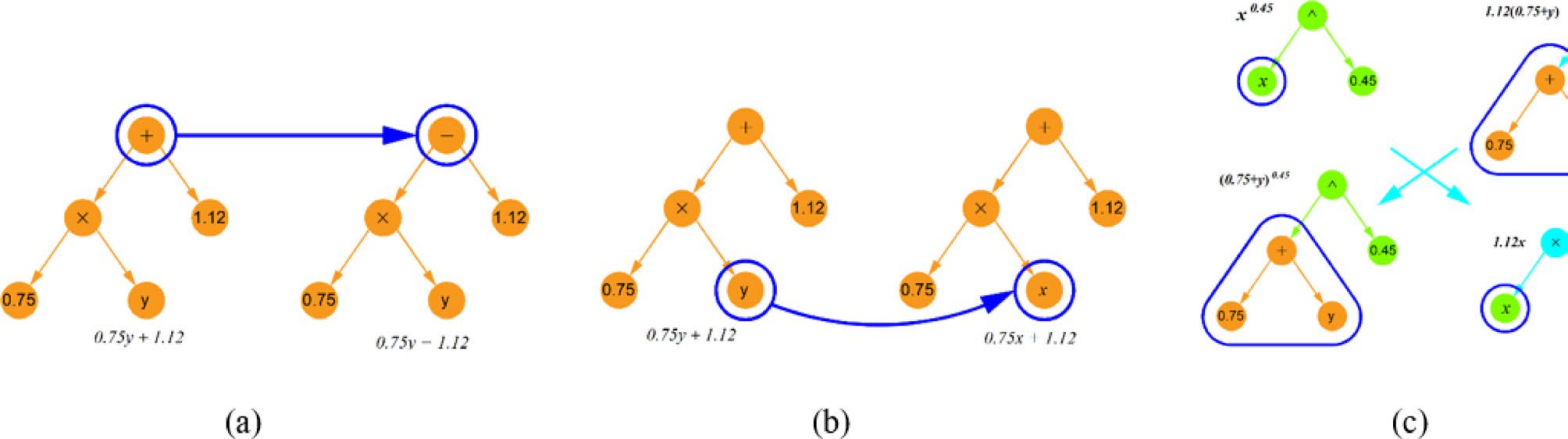
Mutation and crossover operations in SR model. (**a**) A mutation operation on expression tree, (**b**) A mutation operation on input variable, (**c**) A crossover operation between two trees.

**Fig. 4:**
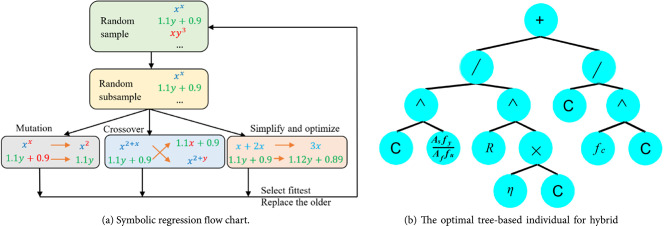
(**a**) Symbolic regression flow chart, (**b**) The optimal tree-based individual for hybrid FRP-steel RC beams, where the variable C is constant, R and $\:\eta\:$ are defined in (Table 5).

The SR algorithm begins by generating an initial population of mathematical expressions composed of operational symbols (e.g., +, −, *,/, ^, etc.) and terminals such as input variables and constants. Each individual expression is structured as a tree. Selection is then performed probabilistically, favoring expressions with superior performance. To avoid excessive complexity, a complexity limit of 30 for the total number of nodes is set, meaning the total number of operators, constants, and variables in any expression cannot exceed this value. Furthermore, overly complex terms, like high-order exponentials (e.g. (•)^(•^•)), are excluded. Selected expressions then undergo mutation or crossover (as shown in Fig. 3) to generate new populations for the next generation. Figure 4a presents the core steps of the SR approach. This evolutionary process is guided by a fitness function, which balances prediction accuracy with simplicity. The fitness function is defined as:

2 $$\:l\left(E\right)={l}_{pred}\left(E\right).\text{exp}\left(\text{f}\text{r}\text{e}\text{c}\text{e}\text{n}\text{c}\text{y}\left[C\left(E\right)\right]\right)$$

where *l*_*pred*_(*E*) and *C*(*E*) define, respectively, the prediction error and the expression complexity *E*, quantified by the total number of nodes in the expression. The frecency [*C*(*E*)] for how often an expression of complexity *C*(*E*) occurs. This measure is crucial for avoiding overcomplicated and redundant expressions, ensuring a balance between minimizing error and maintaining simplicity.

To enhance transparency and interpretability, three dimensionless parameters were engineered from the raw dataset and used as inputs to the PySR symbolic regression model:

3 $$\:p=\frac{{A}_{s}{f}_{y}}{{A}_{f}{f}_{fu}},\:\:\:\:\:\:\:\:\:\:\:\:R=\frac{{f}_{fu}/{E}_{f}}{{\epsilon\:}_{cu}},\:\:\:\:\:\:\:\:\:\:\:\:\:\eta\:=\frac{{A}_{f}{f}_{fu}+{A}_{s}{f}_{y}}{{f}_{c}\left(b{d}_{s}\right)}$$

Here, $\:{\epsilon\:}_{cu}=0.003$ represents the ultimate strain of concrete. The parameter *p* reflects the relative contribution of steel to FRP reinforcement, *R* represents a normalized strain ratio capturing the ductility effect of the FRP system and *η* denotes a dimensionless strength index that balances the reinforcement capacity with the concrete strength. These parameters were engineered from the raw experimental dataset based on physical relevance and domain knowledge, then used as inputs in the PySR symbolic regression algorithm to evolve the final interpretable form presented.

The search for optimal expressions involves numerous iterations, each producing potential solutions. During this process, factors such as model complexity, prediction accuracy, and ease of interpretation are carefully evaluated and refined. By varying key parameters like population size, number of generations, and mutation rate, different equations are generated and assessed, ensuring that the final model offers a balance between simplicity and predictive power. This iterative refinement guarantees that the selected model is both interpretable and accurate. Details of the SR parameters used for generating expressions in this study are summarized in (Table 4).

**Table 4:** The parameters of the SR model used in generating expressions.

Parameters	Value	Parameters	Value
Number of generations	200	Crossover probability	0.066
Total number of populations	40	Tournament size	10
Population size	30	Allowed binary operators	+, *, ^
Maximum length of expressions (total number of nodes)	30	Loss function	MAPE%
Parsimony (factor controls the expression complexity)	0.01	Constraints	{‘^’:(–1,10)}^(a)^
Mutation rate for constants	0.048	Nested constraints	{“^”:{“^”:0}}^(b)^
Mutation rate for operators	0.47	model_selection	Accuracy

(a) The ‘^’:(–1,10) constraint means that the left argument of the power function can exhibit any level of complexity, whereas the right argument is restricted to a maximum complexity of 10 nodes.

(b) Nested constraints govern how operators can be combined or nested. The constraint ‘^’:{‘^’:0} specifies that ‘^’ operator cannot be used inside other ‘^’ operator.

The optimal tree-based model (Fig. 4b) fitted to the training experimental database for hybrid FRP/steel reinforced concrete beams is expressed as:

4 $$\:{m}_{u}=\frac{{M}_{u}}{{A}_{f}{f}_{fu}{d}_{f}+{A}_{s}{f}_{y}{d}_{s}}=\frac{{1.037}^{p}}{{R}^{1.1\eta\:}}+\frac{1}{{{f}_{c}}^{0.47}},\:$$

This formulation is consistent with the trends observed in (Fig. 2). The strength index $\:{m}_{u}$ decreases as steel reinforcement and FRP reinforcement ratios increase. Furthermore, increasing the ratio of steel reinforcement tensile strength $\:{A}_{f}{f}_{fu}$ and FRP reinforcement tensile strength $\:{A}_{f}{f}_{fu}$ will enhance the flexural strength index. On the other hand, the concrete compressive strength ($\:{f}_{c}$) exhibits a mixed effect, as evident in (Fig. 2).

Table 5 summarizes the proposed design, ACI 440.11–22 standards^12^ and GEP model generated by Zhang et al.^11^ for hybrid FRP-steel RC beams. The ACI 440.11–22^12^ guidelines outline two procedures for calculating flexural strength, depending on the controlled section failure modes: There are two failure modes, Tension-Controlled Section Failure and Compression-Controlled Section Failure. In the first failure mode, failure occurs when the FRP bars reach their ultimate tensile strength and rupture, while the steel bars yield and the concrete strain in the compression zone remains below its ultimate strain. While, in Compression-Controlled Section Failure mode, failure occurs due to concrete crushing on the compressive side. Here, the steel bars yield, but the strain in the FRP bars remains below their ultimate tensile strain. Due to the complexity of determining the concrete compressive strain and the neutral axis depth, Zhang et al.^11^ recently have introduced a design equation for GEP model for hybrid FRP-steel RC beams. While the introduced expression demonstrates efficiency and aligns well with experimental results, their formula is overly complex, unit-dependent, and lacks sufficient explanation. This paper introduces a novel model to derive simple, interpretable, and unit-independent expressions for predicting the flexural strength of hybrid FRP-steel RC beams.

**Table 5:** Summary of ACI440.11–22 provisions with the proposed design.

	Formulas
ACI440.11–22^12^	Tension-controlled section $\:\left(0 <\frac{{\rho\:}_{eff,f}}{{\rho\:}_{f,b}}\le\:1\right)$ $\:{\text{c}}_{b}=\left(\frac{{\epsilon}_{\text{c}\text{u}}}{{\epsilon}_{\text{c}\text{u}}+{\epsilon}_{fu}}\right){d}_{0},\:\:{M}_{\text{u}}=\left({A}_{f}{f}_{f\text{u}}+{A}_{s}{f}_{y}\right)\left({d}_{0}-\frac{{\beta\:}_{1}{\text{c}}_{b}}{2}\right)$ Compression-controlled section $\:\left(\frac{{\rho\:}_{eff,f}}{{\rho\:}_{f,b}}>1\right)$ $\:{A}_{f}{f}_{f}+{A}_{s}{f}_{y}=0.85{\beta\:}_{1}{\text{f}}_{c}bc$ $\:{f}_{f}={E}_{\text{f}}{\epsilon\:}_{\text{c}\text{u}}\frac{{\text{d}}_{\text{f}}-\text{c}}{\text{c}}$ $\:{\text{f}}_{f}=\sqrt{\frac{1}{4}{\left(\frac{{A}_{s}{f}_{y}}{{A}_{f}}+{E}_{f}{\epsilon\:}_{cu}\right)}^{2}+{E}_{f}{\epsilon\:}_{cu}\frac{0.85{\beta\:}_{1}{\text{d}}_{f}{\text{b}\text{f}}_{\text{c}}-{A}_{s}{f}_{y}}{{A}_{\text{f}}}}-\frac{1}{2}\left(\frac{{A}_{s}{f}_{y}}{{A}_{f}}+{E}_{f}{\epsilon\:}_{cu}\right)\le\:{f}_{fu}$ $\:{M}_{\text{u}}={A}_{f}{f}_{f}\left({d}_{f}-\frac{{\beta\:}_{1}\text{c}}{2}\right)+{A}_{s}{f}_{y}\left({d}_{s}-\frac{{\beta\:}_{1}\text{c}}{2}\right)$ where $\:{\rho\:}_{s}=\frac{{A}_{s}}{b{d}_{s}},\:\:{\rho\:}_{f}=\frac{{A}_{f}}{b{d}_{f}},\:\:{\rho\:}_{eff,f}={\rho\:}_{s}\frac{{f}_{y}}{{f}_{fu}}+{\rho\:}_{f},\:{\rho\:}_{f,b}=0.85*{\beta\:}_{1}\frac{{f}_{c}}{{f}_{fu}}\frac{{\epsilon\:}_{\text{c}\text{u}}}{{\epsilon\:}_{\text{f}\text{u}}+{\epsilon\:}_{\text{c}\text{u}}},\:{\epsilon\:}_{\text{c}\text{u}}=0.003$ $\:{\beta\:}_{1}=0.65\le\:0.85-0.05\left(\frac{{\text{f}}_{c}-28}{7}\right)\le\:0.85$ $\:{c}_{b}$Depth of compressive zone at balanced condition (mm) $\:c$Depth of compressive zone (mm) $\:d$Beam depth (mm) $\:{d}_{0}$Depth from the extreme compression fier to the center of the hybrid FPR and steel bars (mm) $\:{\epsilon}_{cu}$Ultimate concrete strain in compression $\:{\epsilon}_{cu}=0.003$ $\:{\epsilon}_{fu}$Ultimate strain of FRP bar
Zhang et al.^11^	$\:{M}_{\text{u}}=\text{e}\text{x}\text{p}\left\{{\left(\left(\frac{b+{E}_{f}}{-12.429+{f}_{fu}}\right)*\left({A}_{s}\right)\right)}^{0.25}\right\}+\text{exp}\left\{{\left(b+\left(\frac{{E}_{f}+h+{A}_{f}}{{f}_{fu}^{0.2}}\right)\right)}^{0.25}\right\}+\left(-20.651-\frac{{f}_{c}+{A}_{s}}{{f}_{c}}+\text{exp}\left(-4.917\right)*\left({f}_{fu}+{f}_{y}\right)\right)$
Proposed design	$\:\frac{{M}_{u}}{{A}_{f}{f}_{fu}{d}_{f}+{A}_{s}{f}_{y}{d}_{s}}=\frac{{1.037}^{p}}{{R}^{1.1\eta\:}}+\frac{1}{{{f}_{c}}^{0.47}},\:\:\:\:\:\:\:\:\:\:\:\:\text{w}\text{h}\text{e}\text{r}\text{e}\:p=\frac{{A}_{s}{f}_{y}}{{A}_{f}{f}_{fu}},\:\:\:\:\:\:\:\:\:\:\:\:R=\frac{{f}_{fu}/{E}_{f}}{{\epsilon\:}_{cu}},\:\:\:\:\:\:\:\:\:\:\:\:\:\eta\:=\frac{{A}_{f}{f}_{fu}+{A}_{s}{f}_{y}}{{f}_{c}\left(b{d}_{s}\right)}$

To further highlight the parsimony of our PySR-derived model, its symbolic complexity is compared against Zhang et al.’s GEP form. Our model contains only 13 total expression nodes (operators, variables, and constants) with a maximum tree depth of 4, whereas Zhang et al.’s equation employs 30 nodes and reaches a depth of 6. Moreover, our expression is primary dimensionless and significantly more compact, facilitating both clearer interpretation and easier implementation.

### Performance and results of ML models

**Fig. 5:**
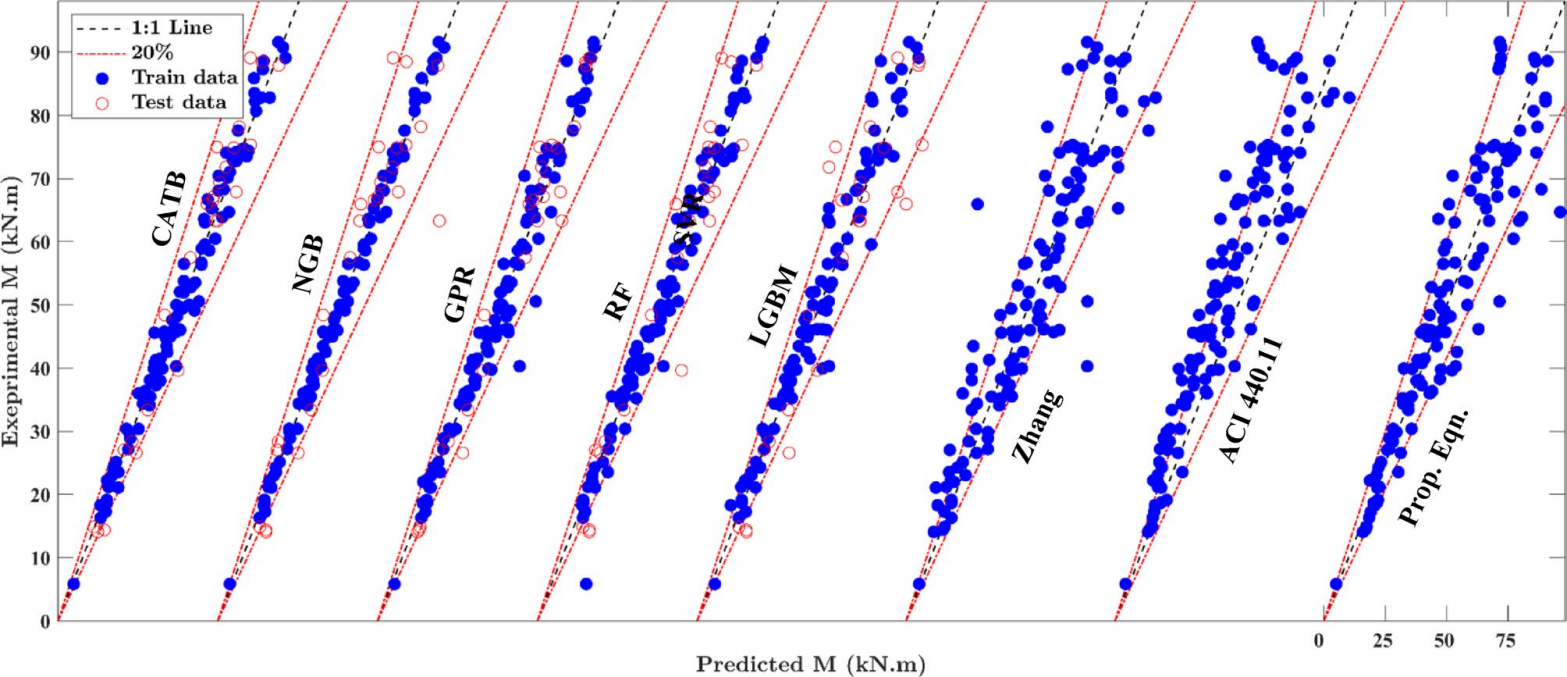
Comparison between experimental and prediction results for the introduced ML models.

**Fig. 6:**
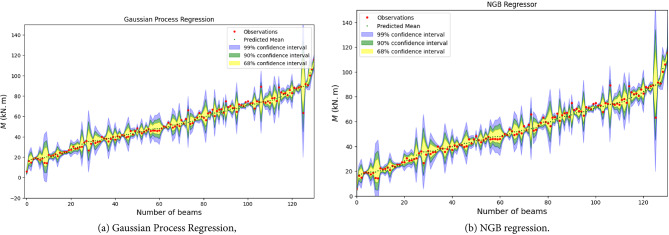
Prediction confidence distribution with a semilog scale on the y-axis for hybrid FRP-steel RC beams. (**a**) Gaussian process regression, (**b**) NGB regression.

This section presents a comprehensive performance evaluation of the machine learning (ML) models developed in this study. Details regarding the ML models, including hyperparameter tuning processes and results, are provided in the supplementary data. Figure 5 illustrates scatter plots comparing experimental and predicted strength values for hybrid FRP-steel RC beams using different ML models on both training and testing datasets. Most data points are closely aligned with the diagonal line, indicating a strong correlation between model predictions and experimental results, which underscores the accuracy and reliability of the developed ML models. Beyond their high predictive accuracy, the GPR and NGBoost models, as shown in Fig. 5, also offer confidence intervals for their predictions, as demonstrated in (Fig. 6). This capability to quantify uncertainty enhances their practical utility by providing confidence levels for prediction results.

Table 6 presents essential metrics for evaluating the performance of the ML models: (1) the mean (µ), which reflects the ratio of actual to predicted values, offering a broad measure of accuracy; (2) the coefficient of variance (CoV), highlighting the variability of predictions compared to the mean; (3) the coefficient of determination (R²), showing how well the model explains the variance in the dependent variable; (4) the root mean squared error (RMSE), which measures the average prediction error, particularly focusing on larger errors; and (5) the mean absolute percentage error (MAPE), which calculates the percentage difference between actual and predicted values. The corresponding formulas for these metrics are outlined as follows:

**Table 6:** Comparison of the developed ML models.

Metrics	Training data			Testing data				All data					
	GPR	NGB	CatB	GPR		NGB	CatB	GPR	NGB	CatB	ACI 440 [12]	Zhang et al. [11]	Prop. design
Mean $\:\mu\:$	0.996	1	0.998	1.027		1.051	1.071	1.002	1.01	1.013	1.18	1.043	1.003
CoV	0.072	0.037	0.058	0.184		0.242	0.263	0.107	0.119	0.138	0.151	0.201	0.139
R^2^	0.975	0.996	0.986	0.905		0.826	0.806	0.902	0.86	0.82	0.918	0.932	0.929
MAPE	5.19	2.69	4.75	11.509		16.302	14.697	6.46	5.43	6.75	20.63	13.91	11.08
RMSE(kN)	3.8	1.5	2.8	25.5		30	33.7	11.9	13.5	15.4	12.5	9.00	9.10

5 $$\:\mu\:=\frac{1}{n}\sum\:_{i=1}^{n}\frac{{y}_{i}}{{\widehat{y}}_{i}},\:\:{R}^{2}=1-\frac{\sum\:_{i=1}^{n}{\left({\widehat{y}}_{i}-{y}_{i}\right)}^{2}}{\sum\:_{i=1}^{n}{\left({y}_{i}-\stackrel{-}{y}\right)}^{2}}\:\:RMSE=\sqrt{\frac{1}{n}\sum\:_{i=1}^{n}{\left({\widehat{y}}_{i}-{y}_{i}\right)}^{2}},\:\:MAPE=\frac{1}{n}\sum\:_{i=1}^{n}\left|\frac{{y}_{i}}{{\widehat{y}}_{i}}-1\right|\times\:100\%$$

Here, $\:{y}_{i}$ and $\:{\widehat{y}}_{i}$ represent the actual experimental and predicted flexural strength values of the *i-*th sample, respectively, $\:\stackrel{-}{y}$ denotes the mean of the experimental results, and *n* is the total number of samples in the database.

The evaluation metrics presented in Table 6 highlight the robust performance of all machine learning (ML) models across hybrid FRP-steel RC beams. The mean *µ*, *R*^*2*^, and a20-index values for GPR, NGBoost (NGB), and CatBoost (CatB) models are close to 1.0, with small CoV, MAPE%, and RMSE values. This indicates a high degree of accuracy and minimal deviation in predictions compared to the experimental results. As noticed, the CoV for these models is less than 0.138, and MAPE% values are lower than 6.75, indicating minimized scattering in the prediction results compared to the experimental results. The GPR model exhibits the best performance with MAPE% values of 5.19 for the training set and 11.51 for the testing set. The NGB and CatB models also perform well, with MAPE% values of 2.69 and 4.75 for training and 16.30 and 14.70 for testing, respectively. It should be noted that the significant performance gap between training and testing results is influenced by the relatively small size of the dataset, with only 26 data points used for testing. This limited sample is more susceptible to the effects of outliers, which may distort performance metrics.

While the GPR model demonstrates superior performance, its black-box nature limits practical application in engineering design, highlighting the need for more interpretable models like the symbolic regression model. When comparing the ML models with the proposed expression, it displays a mean *µ* of 1.003, a CoV of 0.139, and a MAPE% values of 11.08. Although the accuracy of the proposed design is slightly lower than that of GPR, NGB, and CatB, its results are comparable to the remaining ML models. Additionally, the proposed design offers better interpretability, making it more practical for real-world applications, in contrast to the black-box nature of many ML models. In contrast, ACI 440.11–22^12^ and GEP model by Zhang et al.^11^ presents higher errors, with CoV values of 0.151 and 0.201, and MAPE% values of 20.63 and 13.91. These results clearly demonstrate that the proposed design, utilizing symbolic regression, greatly improves accuracy by significantly reducing error indices compared to previous models, making it a more reliable method for predicting the flexural strength prediction of hybrid FRP-steel RC beams.

**Fig. 7:**
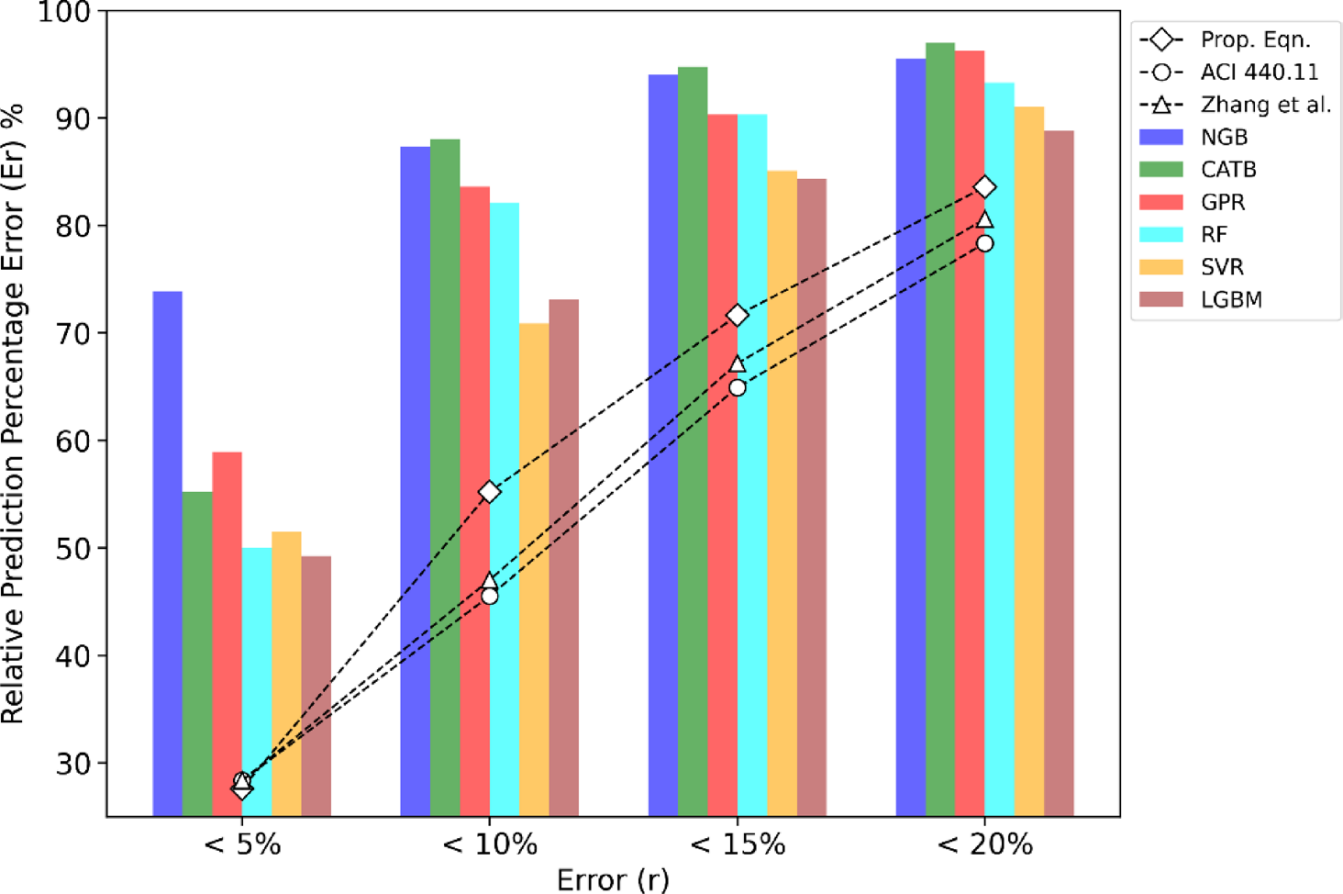
Prediction errors histogram of the established ML models.

Figure 7 demonstrates that most of the introduced ML models outperform the previous models in terms of predictive accuracy, particularly within the 10% error range. The CATB, NGB, and GPR models capture over 83% of experimental samples within the 10% error range, specifically 88%, 87%, and 83%, respectively. This highlights the superior performance of the ML models in predicting the flexural strength of hybrid FRP-steel RC beams. In comparison, the proposed design captures 57% of samples within the 10% error range, whereas ACI 440.11–22 standards, and Zhang model capture 45% and 47%, respectively. While the ML models excel in precision, the proposed design still delivers acceptable results, offering a more practical solution compared to the black-box nature of many ML models.

Beyond accurate prediction, the derived symbolic regression model offers interpretable insights into the mechanics of hybrid FRP-steel RC beams. Due to its concise, primarily dimensionless form, the expression can help identify dominant parameters and their nonlinear interactions, such as the sublinear influence of concrete strength or reinforcement ratios. Its transparency and simplicity make it a strong candidate for developing design-oriented formulations or aiding in the calibration of simplified code-based equations.

### Out-of-distribution (OOD) generalization testing based on input extremes

To assess the generalization capability of the proposed symbolic regression model beyond the core distribution of the training data, an out-of-distribution (OOD) evaluation procedure was performed. Specifically, for each input parameter individually, the dataset was sorted in ascending order based on that parameter. The bottom 10% and top 10% of data points—representing the extreme low and high values—were extracted as the OOD test set, while the remaining middle 80% was used for results comparison. The feature-specific extrapolation testing method introduced is inspired by OOD generalization principles in machine learning (e.g., Arjovsky et al.^41^).

This approach simulates extrapolation behavior by evaluating the model on input regions not observed during training. The model was retrained for each feature-specific split, and the Mean Absolute Percentage Error (MAPE) was computed between predicted and actual values over the 20% extreme set. This procedure was repeated for each input variable to isolate the model’s extrapolation performance along different feature dimensions.

As shown in Table 7, we report three key performance metrics—mean prediction ratio (Mean µ), coefficient of variation (CoV), and mean absolute percentage error (MAPE)—for both the central 80% of the data and the combined lowest/highest 10% extremes across all eight input features, alongside the reference values computed over the full dataset. Overall, the OOD results remain very close to those on the central 80%: mean prediction ratios stay near unity (within ± 6%), CoV and MAPE vary only slightly, and no single feature exhibits a dramatic performance drop. This demonstrates that the introduced symbolic regression model generalizes robustly even at the extreme low and high ends of each input parameter.

**Table 7:** Out-of-Distribution (OOD) generalization testing results.

Metrics	Split	$\:b$	$\:d$	$\:{\rho\:}_{s}$	$\:{\rho\:}_{s}$	$\:{f}_{c}^{{\prime\:}}$	$\:{f}_{y}$	$\:{f}_{fu}$	$\:{E}_{f}$	Ref
Mean $\:\mu\:$	Middle 80%	1.019	1.012	1.013	0.991	1.017	1.01	0.996	1.012	**1.003**
	Outliers: lowest 10% and highest 10%	0.938	0.964	0.963	1.054	0.944	0.973	1.031	0.967	
CoV	Middle 80%	0.139	0.14	0.135	0.133	0.133	0.13	0.145	0.138	**0.139**
	Outliers: lowest 10% and highest 10%	0.114	0.126	0.146	0.148	0.15	0.17	0.107	0.136	
MAPE	Middle 80%	11.37	11.13	10.58	10.29	10.77	10.24	11.68	11.14	**11.08**
	Outliers: lowest 10% and highest 10%	9.84	10.86	13.13	14.36	12.36	14.55	8.58	10.83	

### Feature importance analysis

**Fig. 8:**
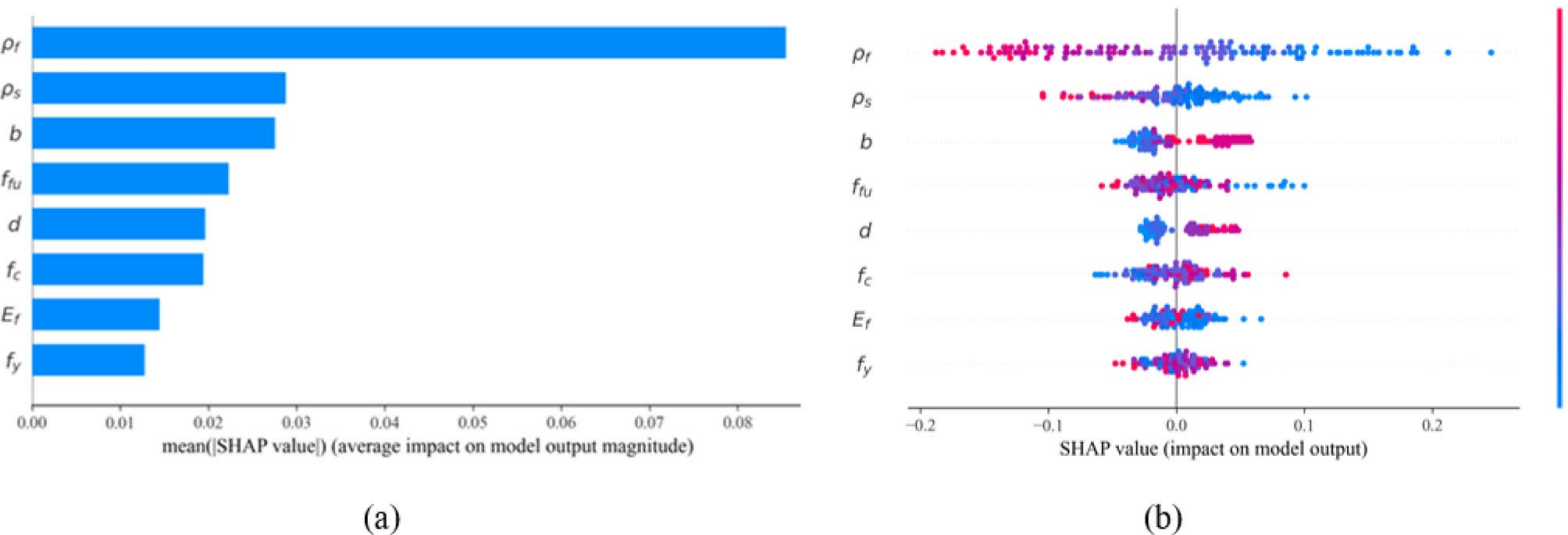
Feature importance analysis for inputs influencing flexural strength index $\:{m}_{u}$. (**a**) Summary plot, (**b**) SHAP feature importance.

Assessing the influence of input parameters on the flexural strength index is essential for the effective design of hybrid FRP-steel reinforced concrete (RC) beams. In this study, the Shapley Additive Explanations (SHAP) method is employed to quantify the contribution of each input variable to the strength index. Figure 8a presents the SHAP summary plot, highlighting both the magnitude and direction of each feature’s impact on model predictions. Figure 8b further illustrates the relative importance of individual features. A positive SHAP value indicates a positive correlation with the strength index, whereas a negative value implies an adverse effect.

Among all input parameters, the steel reinforcement ratio (*ρ*_*s*_%) and FRP reinforcement ratio (*ρ*_*f*_%) emerge as the most influential parameters. Interestingly, higher values of these ratios are associated with a decline in flexural strength index $\:{m}_{u}$, likely due to the brittle behavior observed in over-reinforced RC beams. The remaining features are ranked according to their importance. Notably, beam width (*b*), beam depth (*d*), and concrete compressive strength (*f*_*c*_) show a consistently positive or mixed influence on the strength index. This indicates that increasing cross-sectional dimensions and concrete strength tends to improve the flexural capacity of the beams by enhancing their ductility.

## Conclusions

This study developed two advanced computational approaches—machine learning (ML) and symbolic regression—to predict the flexural strength of hybrid FRP-steel reinforced concrete (RC) beams using a database of 134 experimental data points, with eight key parameters serving as input variables: beam width, beam depth, steel and FRP reinforcement area, concrete compressive strength, steel bar yield strength, ultimate tensile strength of FRP bars, and elasticity modulus of FRP bars. The performance of the proposed ML and symbolic regression models was compared against the ACI 440.11–22^12^ equations, and GEP model by Zhang et al.^11^ was conducted to evaluate the influence of input variables. The following conclusions were drawn:

- Symbolic regression provided a simplified, interpretable mathematical equation to predict the flexural strength of hybrid FRP-steel RC beams. The introduced equation not only enhances predictive accuracy but also offers physical insights into the relationship between input variables and flexural strength, bridging the gap between data-driven models and traditional engineering approaches.
- The ML models and symbolic regression demonstrated strong agreement with experimental results. The GPR model achieved MAPE% values of 0.992 (training) and 0.979 (testing), while the symbolic regression model achieved MAPE% of 0.979 (training) and 0.939 (testing). These results confirm the reliability and accuracy of both approaches in predicting flexural strength.
- While the GPR model demonstrates superior performance, its black-box nature limits practical application in engineering design, highlighting the need for more interpretable models like the symbolic regression model.
- The proposed ML and symbolic regression models outperformed the ACI 440.11–22 equations, and GEP model by Zhang et al., exhibiting lower mean absolute error (MAE), mean absolute percentage error (MAPE), and root mean squared error (RMSE), as well as higher R^2^ values. This highlights the superiority of data-driven approaches over traditional design guidelines.
- SHAP analysis showed that larger cross-sectional dimensions and higher concrete strength improve the flexural performance of hybrid FRP-steel RC beams, whereas increasing the reinforcement ratios will negatively impact the flexural strength.

Future research should aim to expand the experimental database to improve model generalization and reduce overfitting risks. Additionally, exploring hybrid modeling techniques that combine physics-informed constraints with machine learning could further enhance interpretability and robustness. Moreover, investigating the capacity of hybrid beams composed of Engineered Cementitious Composite (ECC) and reinforced concrete (RC) with hybrid FRP-steel reinforcement presents a promising avenue, given ECC’s superior tensile strength and crack control capabilities.

## Electronic supplementary material

Below is the link to the electronic supplementary material.

Supplementary Material 1

## Data Availability

All data generated or analyzed during this study are included in this published article and available in a public repository: https://github.com/kmegahed/Hybrid-FRP-Steel-Reinforced-Beams-Machine_learning.
